# An effective strategy for recapitulating N-terminal heptad repeat trimers in enveloped virus surface glycoproteins for therapeutic applications†
†Electronic supplementary information (ESI) available: General materials, methods, and the details. See DOI: 10.1039/c5sc04046a


**DOI:** 10.1039/c5sc04046a

**Published:** 2015-12-03

**Authors:** Wenqing Lai, Chao Wang, Fei Yu, Lu Lu, Qian Wang, Xifeng Jiang, Xiaoyu Xu, Tianhong Zhang, Shengming Wu, Xi Zheng, Zhenqing Zhang, Fangting Dong, Shibo Jiang, Keliang Liu

**Affiliations:** a State Key Laboratory of Toxicology and Medical Countermeasures , Beijing Institute of Pharmacology & Toxicology , 27 Tai-Ping Road , Beijing , 100850 , China . Email: keliangliu55@126.com ; Fax: +86-10-68211656 ; Tel: +86-10-6816-9363; b Key Laboratory of Medical Molecular Virology of Ministries of Education and Health , Shanghai Medical College , Shanghai Public Health Clinical Center , Fudan University , Shanghai 200032 , China . Email: shibojiang@fudan.edu.cn ; Fax: +86-21-54237465 ; Tel: +86-21-54237673; c Lindsley F. Kimball Research Institute , New York Blood Center , New York , NY 10065 , USA; d National Center of Biomedical Analysis , 27 Tai-Ping Road , Beijing , 100850 , China

## Abstract

Sequestering peptides derived from the N-terminal heptad repeat (NHR) of class I viral fusion proteins into a non-aggregating trimeric coiled-coil conformation remains a major challenge. Here, we implemented a synthetic strategy to stabilize NHR-helical trimers, with the human immunodeficiency virus type 1 (HIV-1) gp41 fusion protein as the initial focus. A set of trimeric scaffolds was realized in a synthetic gp41 NHR-derived peptide sequence by relying on the tractability of coiled-coil structures and an additional isopeptide bridge-tethering strategy. Among them, (N36M)_3_ folded as a highly stable helical trimer and exhibited promising inhibitory activity against HIV-1 infection, exceptional resistance to proteolysis, and effective native ligand-binding capability. We anticipate that the trimeric coiled-coil recapitulation methodology described herein may have broader applicability to yield NHR trimers of other class I enveloped viruses and to prepare helical tertiary structure mimetics of certain natural protein–protein interactions for biomedical applications.

## Abstract 2

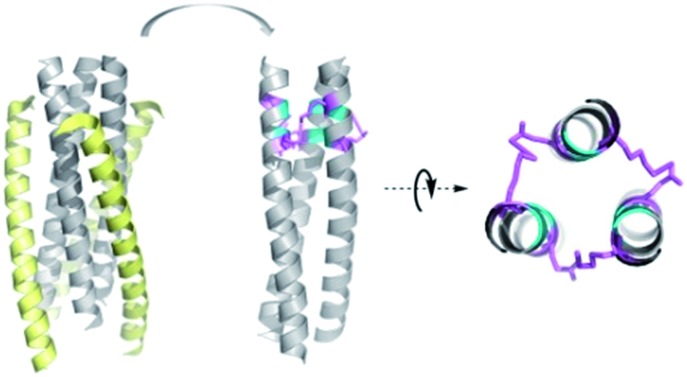
We report an efficient strategy to recapitulate NHR α-helical trimers in the HIV-1 membrane fusion protein as promising antiviral therapeutics.

## Introduction

**Fig. 1:**
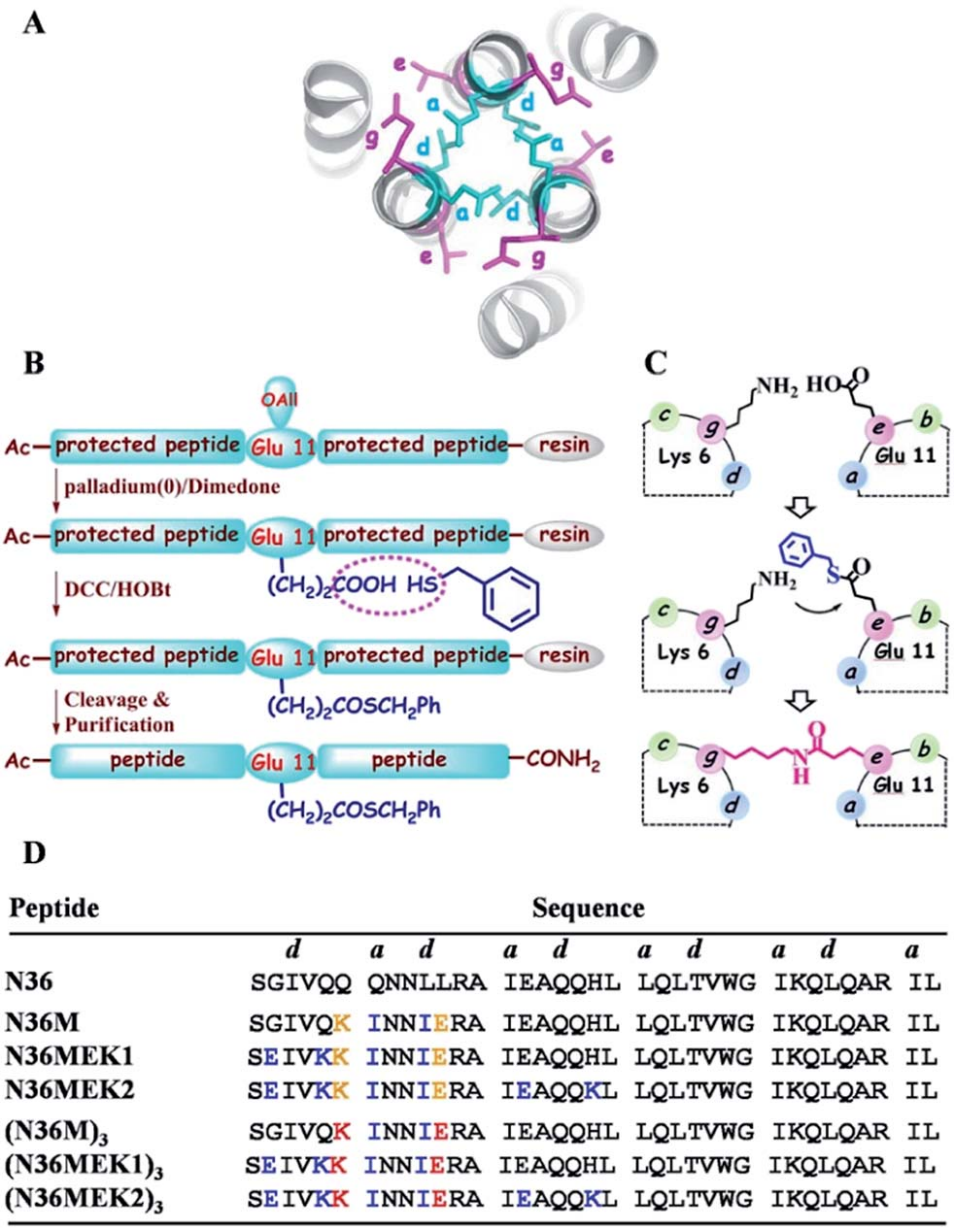
(A) Top view of the structure of HIV-1 gp41 6-HB (PDB: 1AIK). (B) Strategy for the preparation of thioester-modified peptides. (C) Schematic representation of isopeptide bond formation *via* an inter-helical acyl transfer reaction. For clarity, only one of the three symmetrical active sites is shown. (D) The sequences of our designed N36 derivatives. The inter-helical *i* to *i*′ + 5 ionic interactions are shown in yellow. Isopeptide bonds are formed between Lys-6 and Glu-11 (in red). These peptides have an acetyl group at the N-terminus and carboxyamide at the C-terminus.

One of the hallmarks of class I viral fusion proteins on the periphery of enveloped viruses such as human immunodeficiency virus type 1 (HIV-1), Middle East respiratory syndrome coronavirus (MERS-CoV), and Ebola virus is the triggered formation of an α-helical trimer-of-hairpins motif that is a critical prelude to the fusion between the virus and the target cell membrane.^1^ The trimer-of-hairpins of HIV-1 is arguably the most typical example of such a coiled-coil six-helical bundle (6-HB), where the N-terminal heptad repeats (NHRs) of the viral envelope glycoprotein (Env) gp41 subunit form the central trimeric coiled-coil inner core, and three C-terminal heptad repeats (CHRs) pack obliquely in an antiparallel manner (Fig. 1A).^2^ Previous studies have indicated that an extended pre-hairpin intermediate exists, in which the trimeric NHR coiled-coil or the CHR motif is exposed.^3^ Inhibition of the transitions and folding of the pre-hairpin intermediates into their fusogenic 6-HBs has significant biomedical potential in interrupting the process of membrane fusion and stopping viral infection.^4^ The first proof of concept came from the study of a peptide corresponding to the HIV-1 gp41 CHR region, *i.e.*, T20 (Enfuvirtide, Fuzeon), which was approved by the U.S. FDA as the first HIV-1 fusion inhibitor.^5^ The paradigm established by T20 for HIV-1 has launched the identification of a new line of analogous CHR peptide-based fusion inhibitors for other class I viral fusion proteins, with promising antiviral activity.^6^–^9^ In sharp contrast, peptides derived from the NHR region of the fusion proteins are generally much less potent inhibitors than CHR peptides, and their development as antiviral agents has been hampered because linear NHR peptides on their own aggregate under physiological conditions and cannot automatically form their naturally occurring trimeric coiled-coil structure, a prerequisite for interaction with the complementary CHR region.^9^–^11^ Therefore, efforts have been directed toward the preparation of stable and soluble N-trimer mimetics. These have included the construction of several chimeric molecules by introducing an exogenous trimerization motif to a portion of the N-helix and further stabilization of these trimeric oligomers through disulfide bonds.^12^–^16^ However, these modifications suffer from extra-large auxiliary protein domains, which may attenuate the antiviral activity of the NHR-trimer mimetics,^10^ and if used as antigens, the nonrelevant portion of the peptide chimera might induce undesired immune recognition.^17^ Moreover, the exogenous motifs might divert the real conformation of the NHR trimer in the prefusogenic state, thus thwarting their application as drug targets to screen small molecules^18^ or identify therapeutics *via* mirror image phage display.^19^

Isopeptide bonds present in a range of bacterial pilus structures give extraordinary stability to these capsids in the face of severe mechanical, chemical, and proteolytic stress.^20^ Seminal work investigating the stability of *de novo* designed coiled-coil interactions suggests that isopeptide bonds in these *de novo* coiled-coil assemblies add exceptional resistance to unfolding.^21^ Herein, we report a synthetic approach to construct NHR trimeric coiled coils homologous with naturally occurring protein sequences as antiviral therapeutics. This approach takes advantage of several rules-of-thumb for coiled-coil trimers and translates the isopeptide bridge-tethering structure from a designed simple sequence to the more challenging native protein coiled coil. We chose NHR peptide-based HIV-1 fusion inhibitors as templates for our study, based on their low capacity to sequester into a non-aggregating helical trimer conformation, which is a true reflection of the practical challenges for broader recapitulation of NHR trimers of class I fusion proteins.

## Results and discussion

### Design

In the HIV-1 gp41 6-HB, three N36 peptides form the central coiled-coil core using hydrophobic residues at the *a*–*d* positions in their helices for self-association and the hydrophobic *e*–*g* residues to facilitate binding with the *a*–*d* residues of the target C34 peptide (Fig. 1A).^22^ Accumulated evidence has shown that the packing interactions of Ile residues in the hydrophobic core favor trimeric coiled-coil assembly and that inter-helical (*g–e*′ and *g*′*–e*) or intra-helical (*i* to *i* + 3 and *i* to *i* + 4) ionic interactions are crucial for controlling protein folding and stability.^23^ According to computational modeling and these design principles, three N36 derivatives, *i.e.*, N36M, N36MEK1, and N36MEK2, were designed to facilitate N-peptide trimerization (Fig. 1D). In the design of N36M, the 7th residue Gln and the 10th residue Leu of N36, located at positions *a* and *d*, respectively, were replaced with Ile residues. Then, the 6th residue Gln at the *g* position and the 11th residue Leu at the *e* position of N36 were further substituted by positively and negatively charged residues, respectively, to form favorable interhelical *i* to *i*′ + 5 electrostatic interactions. Based on the structure of N36M, N36MEK1 and N36MEK2 were designed so that Glu–Lys intra-strand salt bridges were introduced at *i* to *i* + 3 or both *i* to *i* + 3 and *i* to *i* + 4 positions of the helical conformation, respectively, to enhance stability of the coiled-coil assemblies.

Furthermore, we envisioned that replacement of the weak inter-helical ionic bonds at the *g*–*e*′ positions with isopeptide bridges would offer an attractive option for stabilizing the trimeric coiled-coil conformation of our engineered N36 derivatives. Consequently, the side chains of the 11th residue Glu of the N36 mutants described above were modified with a benzyl thioester (Fig. 1B), and the resulting peptides, N36M(SBn), N36M(SBn)EK1, and N36M(SBn)EK2, were used as acyl transfer intermediates to generate the covalently stabilized trimeric NHR oligomers, *i.e.*, (N36M)_3_, (N36MEK1)_3_, and (N36MEK2)_3_, *via* an acyl transfer reaction (Fig. 1C and D).

### Unbridged N36-mutants self-assemble into trimeric coiled-coils

The N36 mutants were first analyzed using sedimentation velocity analysis (SVA) to confirm trimer formation. N36MEK2 and N36MEK1 had sedimentation coefficients of 1.54 s and 1.29 s, corresponding to 13.4 kDa and 10.5 kDa, respectively, agreeing with the theoretical molecular masses of the N36MEK2 trimer (12.7 kDa) and the N36MEK1 trimer (12.7 kDa). Strikingly, with the optimization of only four residues, N36M mostly displayed a trimeric form, as shown by a single peak using SVA at 1.61 s, with an obtained molecular mass of 13.4 kDa (Table S1 and Fig. S1, ESI†). Next, we examined the nature and stability of the secondary structure in the trimeric NHR oligomers using CD spectroscopy. The relative helicity of peptides is typically extrapolated by the mean residue ellipticity at 222 nm. The N36 peptide formed partial helical structures with 27% helicity. Notably, the CD spectra of these trimeric assemblies were consistent with a dramatically improved α-helical content, ranging from 62% to 67%. The thermal unfolding transition (*T*_m_) for N36M, N36MEK1, and N36MEK2 was determined to be 57 °C, 61 °C, and 67 °C, respectively, suggesting that stabilization of individual helices contributes significantly to the helical trimer stability (Table S1, ESI†). In addition, their *T*_m_ values were dependent on peptide concentration, further demonstrating that they were self-associating species (Fig. S2, ESI†). Together, these CD and SVA data are consistent with N36M, N36MEK1, and N36MEK2 folding to form thermally stable trimeric coiled-coils as designed.

### Isopeptide bridge-tethered trimeric coiled coils are successfully constructed *via* an inter-helical acyl transfer reaction

**Fig. 2:**
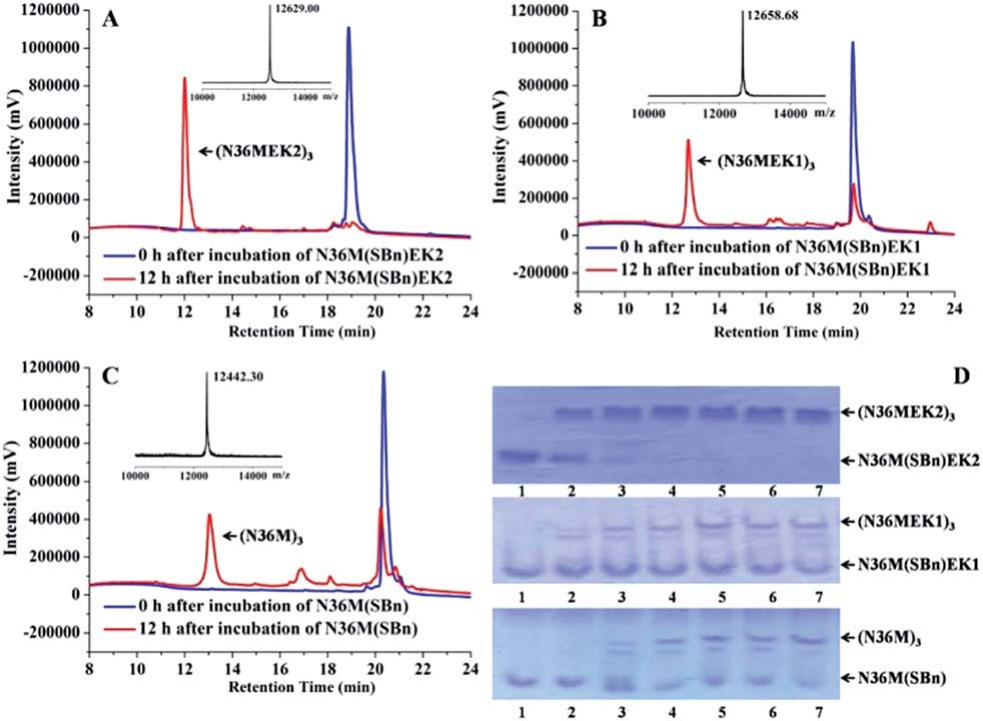
(A) RP-HPLC traces for the acyl transfer reaction of N36M(SBn)EK2 at *t* = 0 and 12 h. The observed MALDI-TOF mass for the product is 12 629.00 Da (calcd: 12 630.18 Da). Lys–Glu ligation of (B) N36M(SBn)EK1 and (C) N36M(SBn) at *t* = 0 and 12 h. The observed MALDI-TOF mass for the product is 12 658.68 Da (calcd: 12 657.02 Da) and 12 442.30 Da (calcd: 12 440.76 Da), respectively. Reactions contained 500 μM thioester-modified peptides in PBS/H_2_O/CH_3_CN (3 : 5 : 2 v/v) at room temperature. (D) SDS–PAGE analysis of the isopeptide bond formation at 0 h (lane 1), 4 h (lane 2), 8 h (lane 3), 12 h (lane 4), 16 h (lane 5), 20 h (lane 6), and 24 h (lane 7).

Subsequently, isopeptide bridge-tethered trimeric coiled coils were constructed *via* an inter-helical acyl transfer reaction (Fig. 2). Reverse-phase (RP)-HPLC showed a nearly complete reaction of N36M(SBn)EK2 and the appearance of a new peak after 12 h. Based on MALDI-TOF-MS analysis, the peak was assigned to the transfer product (N36MEK2)_3_. The interchain acyl transfer reaction was further confirmed by tricine-sodium dodecyl sulfate–polyacrylamide gel electrophoresis (Tricine–SDS–PAGE). Samples were taken from the reaction mixture at different times and stored at –20 °C for PAGE analysis. Consistent with the RP-HPLC analysis, we also witnessed the disappearance of N36M(SBn)EK2 and the appearance of new bands with lower mobility after 4 h of reaction time. As evidenced by RP-HPLC and SDS-PAGE, neither N36M(SBn)EK1 nor N36M(SBn) disappeared after incubation for 12 h, consistent with our previous data, indicating a positive correlation between oligomer stability and acyl transfer rates. To test the specificity of the acyl transfer reaction in N36M(SBn)EK2 and N36M(SBn)EK1, control peptides were generated in which the reactive Lys and its upstream Lys residues were mutated to Arg (Table S2, ESI†). No reaction was observed in the absence of an active site acyl acceptor, which indicated that the benzyl thioester-modified Glu at the *e* position specifically interacts with the 6th residue Lys in the adjacent N-helix at the *g* position, rather than the 5th residue Lys at the *f* position (Fig. S3, ESI†).

### Isopeptide bond-tethered N-trimers exhibit exceptional resistance to thermal denaturation

**Fig. 3:**
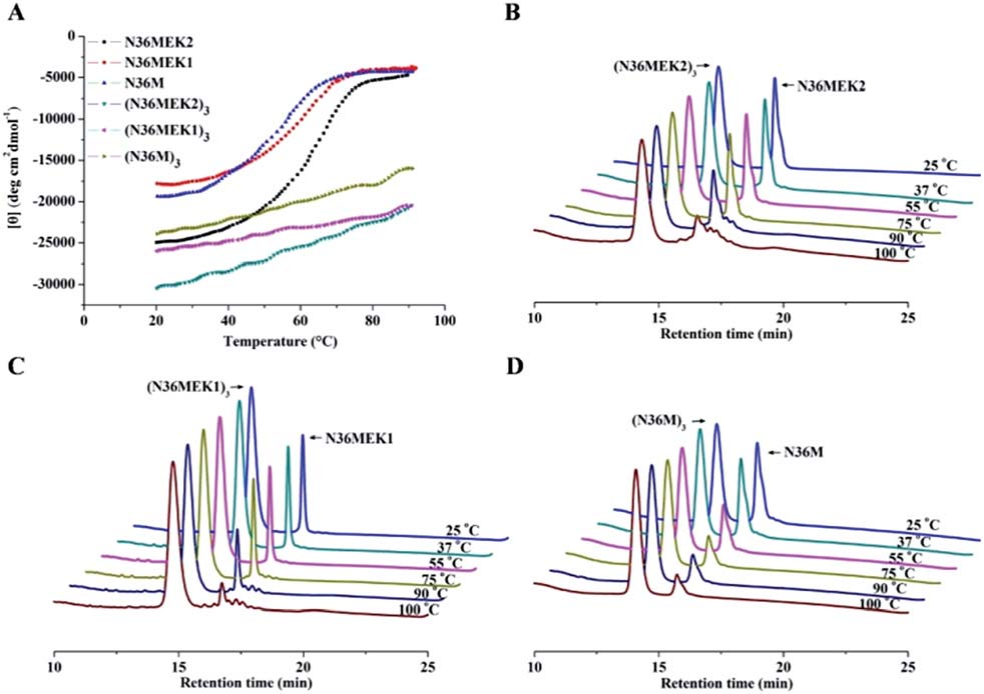
(A) The thermostability of the NHR-trimers at a concentration of 10 μM in PBS was determined using CD spectroscopy. (B)–(D) Increased thermal tolerance of isopeptide bridge-tethered N-trimers. A mixture of crosslinked N-trimers and their uncrosslinked counterparts (1 : 3 molar ratio) was heated at the indicated temperature for 10 min, centrifuged, and the supernatant was analyzed using RP-HPLC.

Next, the trimer states of (N36M)_3_, (N36MEK1)_3_, and (N36MEK2)_3_ were verified using SVA (Table S1, ESI†). CD spectra showed that (N36MEK2)_3_, (N36MEK1)_3_, and (N36M)_3_ formed typical α-helical structures and that the α-helical contents were 100%, 89%, and 82%, respectively, dramatically higher than those of N36MEK2, N36MEK1, and N36M. Strikingly, the covalently stabilized trimeric N-peptides exhibited much higher melting temperatures (*T*_m_ > 90 °C) than those of the unbridged N36-mutants (Table S1, ESI†). To test the effect of the isopeptide bridge-tethering on the thermal stability of the helical trimer, we incubated a mixture of crosslinked N-trimers and their uncrosslinked counterparts over a wide range of temperatures, in order to eliminate interference originating from different protease contaminants in the preparations. A much greater loss of the uncrosslinked N-trimers was noted at 90 °C. On the contrary, the isopeptide bond-tethered trimers were not lost from solution at any of the temperatures tested (Fig. 3).

### Isopeptide bridge stabilized N-trimers are potent inhibitors against HIV-1 Env-mediated cell–cell fusion and infection by laboratory-adapted and clinical HIV-1 strains

To measure the inhibitory activities of these trimeric N-helices, we applied an HIV-1 Env-mediated cell–cell fusion assay. In our assay, N36 displayed moderate inhibitory activity, with a 50% effective concentration (EC_50_) value of 920 ± 84 nM (Table 1). In comparison with N36, N36M, N36MEK1, and N36MEK2 showed no significantly improved inhibitory activity, despite folding into trimeric coiled coils. Strikingly, stabilizing these N-trimers with intermolecular isopeptide bonds dramatically increased their inhibitory activities. (N36M)_3_, which has a sequence that is remarkably similar to that of the naturally occurring gp41 NHR sequence, was the most effective inhibitor of HIV-1 Env-mediated cell–cell fusion, with an EC_50_ value of 4 ± 0.4 nM, similar to the clinically used HIV-1 fusion inhibitor T20, and more potent than (CCIZN17)_3_, a representative chimeric N-peptide fusion inhibitor with high activity.^15^ Both (N36MEK1)_3_ and (N36MEK2)_3_ also exhibited promising inhibitory potencies. Next, we tested the inhibitory activities of our isopeptide bond-tethered N-trimers on the replication of laboratory-adapted and primary HIV-1 strains. A good correlation between the inhibition of HIV-1 Env-mediated cell–cell fusion and inhibition of HIV-1 infection was observed (Tables 1 and 2). Furthermore, all the N-trimers displayed no or low cytotoxicity toward MT-2 cells (Table 1). The observation of subnanomolar anti-HIV-1 activity of these stabilized N-trimers suggests that they are excellent mimics of the NHR region of the gp41 pre-hairpin intermediate and are suitable for future study as drug candidates.

**Table 1:** Anti-HIV-1 activity and cytotoxicity^*a*^

Compound	EC_50_ (nM) for inhibiting			CC_50_ (μM)
	Cell–cell fusion	HIV-1_IIIB_ infection	HIV-1_BaL_ infection	
N36M	1475 ± 73	>10 000	>10 000	>50
N36MEK1	634 ± 69	453 ± 82	698 ± 22	>50
N36MEK2	611 ± 106	317 ± 76	289 ± 85	>50
(N36M)_3_	4.0 ± 0.4	2.67 ± 0.1	8.6 ± 3.1	>50
(N36MEK1)_3_	59.0 ± 8.7	11.2 ± 4.9	74.3 ± 6.5	17.3 ± 1.6
(N36MEK2)_3_	27.1 ± 2.5	4.70 ± 0.6	20.4 ± 2.5	18.9 ± 2.6
N36	920 ± 84	>25 000	>16 000	>50
(CCIZN17)_3_	10.6 ± 1.5	12.1 ± 3.6	5.2 ± 2.0	18.3 ± 1.5
T20	1.2 ± 0.01	57.8 ± 3.2	34.1 ± 9.4	>20

^*a*^Peptides were tested in triplicate, and the data are presented as the mean ± standard deviation.

**Table 2:** Inhibitory activity of peptides on infection by primary HIV-1 isolates^*a*^

Compound	EC_50_ (nM) for inhibiting		
	US4/GS 007 (B, R5)	93IN101 (C, R5)	92TH009 (A/E, R5)
(N36M)_3_	9.4 ± 1.2	5.9 ± 2.0	1.5 ± 0.1
(N36MEK1)_3_	29.2 ± 11.2	20.1 ± 5.6	14.5 ± 4.1
(N36MEK2)_3_	7.1 ± 2.5	5.2 ± 0.9	1.5 ± 0.6
T20	20.4 ± 2.9	22.1 ± 0.9	19.0 ± 0.5

^*a*^Peptides were tested in triplicate, and the data are presented as the mean ± standard deviation.

### Covalently stabilized N-trimers are potent against T20-resistant HIV-1 strains and resistant to proteolysis

**Fig. 4:**
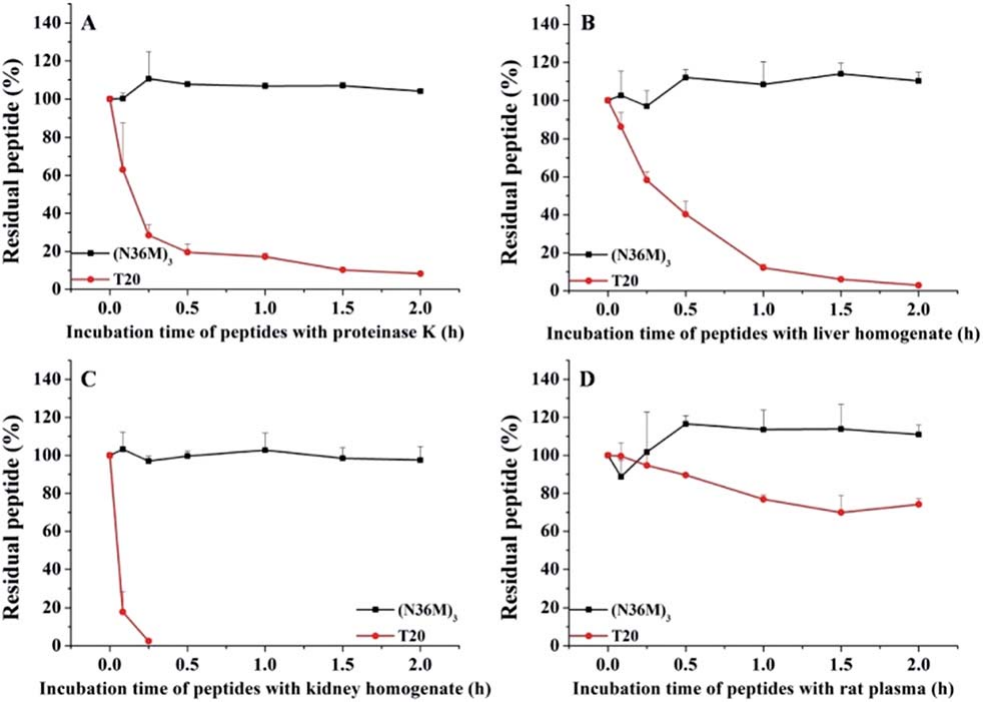
Metabolic stability of (N36M)_3_ and T20 (10 μg mL^–1^) in (A) proteinase K solution, (B) liver homogenate, (C) kidney homogenate, and (D) rat plasma.

The use of the T20 peptide for the treatment of HIV/AIDS patients is limited by increasing resistance to it and its high sensitivity to proteolytic enzymes in the blood.^13^ In our present work, the isopeptide bond-stabilized N-trimers all potently inhibited a series of T20-resistant strains with EC_50_ values ranging from 4.9 nM to 126 nM (Table S3, ESI†). We and others have previously shown that most of the T20-resistant HIV-1 variants contain mutations in the “GIV” motif (amino acids GIVQQQNNLL) located in the gp41 NHR region, which is the main target site of T20 and some other C-peptides.^24^ N-trimer-based HIV-1 fusion inhibitors inhibit HIV-1 entry by targeting the gp41 CHR region. The mutations in the viral gp41 NHR region are not expected to affect the binding of the N-trimer-based fusion inhibitors with the viral gp41 CHR region. Therefore, these well-folded N-trimer-based fusion inhibitors have the advantage over the C-peptide fusion inhibitors because they do not target the “GIV” motif-containing NHR region, thus avoiding cross-drug resistance with T20 and other CHR-based anti-HIV-1 agents. In addition, (N36M)_3_ exhibited dramatically increased *in vitro* metabolic stability over T20 in proteinase K (a broad-spectrum serine proteinase) solution, liver and kidney homogenates, as well as in rat plasma, suggesting that it is a good potential drug candidate for further development (Fig. 4).

From a drug discovery perspective, compounds that possess high potencies of action, relatively few off-target side effects, and optimal ADME (absorption, distribution, metabolism and excretion) properties are nominated as ideal candidates for further development.^25^ Given their tight and specific target-binding, peptides represent an excellent starting point for the design of potential therapeutics. Harnessing bioactive peptides, *e.g.*, T20, to target the coiled coil domain assembly in viral fusion for developing highly selective and potent antiviral agents with minimal systemic toxicity has been successful in clinical application. Unfortunately, T20 has only been relegated to a salvage treatment option, mostly due to accelerated emergence of T20-resistant HIV-1 variants, rapid proteolysis, and high cost of manufacture. The proteolytic shortcoming renders a dosage of 90 mg of T20 subcutaneously twice daily, resulting in increased cost of treatment and poor patient compliance. In our present work, isopeptide bridge-tethered NHR trimeric coiled-coils are totally different species compared with T20 in structure and binding target, thus effectively overcoming HIV drug resistance to T20. Although the cost of producing isopeptide bond-tethered N-trimers would be slightly higher than T20, their much higher resistance to proteolytic degradation should help to prolong the existence of the drug molecules in the circulatory system, which would dramatically decrease the cost of the treatment and improve treatment compliance and patients’ quality of life. It is worth noting that the inhibition of protein–protein interactions has been widely regarded as a challenging task due to their extensive flat and large interfacial areas, as well as noncontiguous binding regions.^26^ Compared with chimeric N-peptides, our recapitulated gp41 NHR helical trimers in the absence of auxiliary protein domains enable more interactions with the target that generate more effective antiviral activity. In addition, the isopeptide bond-tethered N-trimer mimetics may outperform disulfide-bonded chimeric N-peptides in terms of proteolytic stability because disulfides have a possibility to be cleaved *via* the extracellular thiol-containing molecules and oxido-reductases.^27^ Taken together, our constructed gp41 NHR-trimer mimetics have good potential in developing effective, safe, and long-lasting HIV-1 fusion inhibitors.

### Binding of covalently stabilized N-trimers to their complementary gp41 CHR domain

**Fig. 5:**
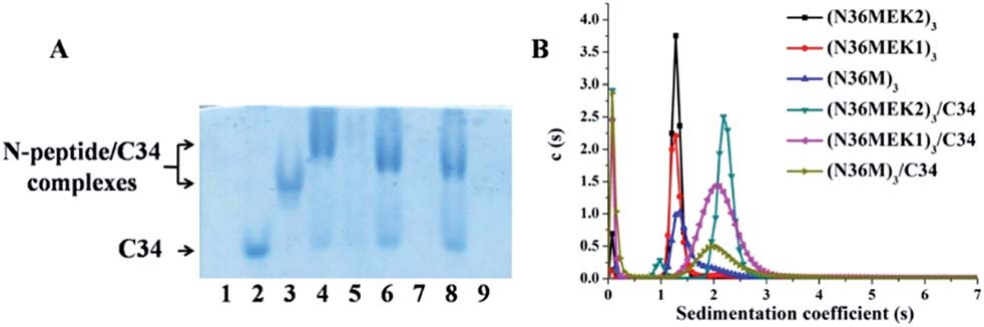
(A) N-PAGE analysis of N-peptides, C34, and their complexes. Lane 1: N36, lane 2: C34, lane 3: C34 + N36, lane 4: (N36MEK2)_3_ + C34, lane 5: (N36MEK2)_3_, lane 6: (N36MEK1)_3_ + C34, lane 7: (N36MEK1)_3_, lane 8: (N36M)_3_ + C34, lane 9: (N36M)_3_. (B) SVA of N-peptides and the N-peptide/C34 mixture.

To test the ability of our stabilized NHR-trimer mimetics to effectively bind their native CHR ligand, native (N)-PAGE, size-exclusion HPLC (SE-HPLC), CD, and SVA were performed. In N-PAGE, covalently stabilized N-peptides do not appear as a result of their net positive charge, similar to other NHR-peptides.^28^ As observed for C34/N36, all stabilized N-trimers formed stable complexes with the target C34 peptide, as evidenced by new bands migrating at a lower rate compared with the C-peptides, concomitant with the near disappearance of the C34 bands (Fig. 5A). The specific interactions between these N-trimers and HIV-1 gp41 CHR were further confirmed using SE-HPLC (Fig. S4, ESI†). In addition, peptide–peptide interactions can also be studied using CD by comparing the spectrum of their mixture (Spec_N+C_) with that of the mathematical sum of the isolated peptides (Spec_N_ + Spec_C_). A difference between the signals indicates an interaction between these peptides.^29^ As shown in Fig. S5,† the covalently stabilized N-trimers interacted with C34 to form complexes that had an increased α-helicity, in comparison to the corresponding Spec_N_ + Spec_C_ value, further confirming the N-PAGE and SE-HPLC results. Finally, the heterogeneous 6-HB states of these stabilized N-trimers with the C34 peptide were ascertained by SVA (Fig. 5B). These combined data indicate that our engineered NHR-trimer mimetics are able to associate with the gp41 CHR region to form heterogeneous 6-HBs that inhibit HIV-1 entry into the target cell.

## Conclusions

In conclusion, we report an effective strategy to construct mimetics of the HIV-1 gp41 NHR trimeric coiled-coil, containing no exogenous trimerization scaffolds. Our study reveals that these NHR trimeric coiled coils homologous with naturally occurring protein sequences have extraordinary thermostability, which contributes positively to their promising inhibition of HIV-1 fusion against a broad spectrum of laboratory-adapted and primary isolates, including those resistant to clinically used T20. In addition, their tertiary structural integrity makes them less sensitive to proteolysis compared to the unstructured peptide T20. Given the common features underlying the class I fusion proteins, our coiled-coil recapitulation methodology shows a high potential for producing NHR-trimer mimetics for other class I enveloped viruses. Moreover, this approach could be extendable to the isolation of certain α-helical trimers for basic scientific and therapeutic applications based on the ubiquitous helical protein–protein interactions found in nature.

## Supplementary Material

Supplementary information
