# Supplementary material for: An effective strategy for recapitulating N-terminal heptad repeat trimers in enveloped virus surface glycoproteins for therapeutic applications
Source: Chem Sci. 2015 Dec 3;7(3):2145–50. doi: 10.1039/c5sc04046a (PMC5968561; doi:10.1039/c5sc04046a)
Supplement: Supplementary file 1 [file SC-007-C5SC04046A-s001.pdf]

Electronic Supplementary Material (ESI) for Chemical Science.
This journal is © The Royal Society of Chemistry 2015

Supporting Information

An Effective Strategy for Recapitulating N-terminal Heptad Repeat Trimers in Enveloped Virus Surface Glycoproteins for Therapeutic Applications

Wenqing Lai,‡[a] Chao Wang,‡[a] Fei Yu,‡[b] Lu Lu,[b] Qian Wang,[b] Xifeng Jiang,[a] Xiaoyu Xu,[a] Tianhong Zhang,[a] Shengming Wu,[d] Xi Zheng,[a] Zhenqing Zhang,[a] Fangting Dong,[d] Shibo Jiang*[bc] and Keliang Liu*[a]

[a] Beijing Institute of Pharmacology & Toxicology, 27 Tai-Ping Road, Beijing, 100850, China;
[b] Key Laboratory of Medical Molecular Virology of Ministries of Education and Health, Shanghai Medical College and Institute of Medical Microbiology, Fudan University, Shanghai 200032, China;
[c] Lindsley F. Kimball Research Institute, New York Blood Center, New York, NY 10065, USA;
[d] National Center of Biomedical Analysis, 27 Tai-Ping Road, Beijing, 100850, China;
‡These authors contributed equally to this work.

Corresponding Authors:

K.L.: State Key Laboratory of Toxicology and Medical Countermeasures, Beijing Institute of Pharmacology & Toxicology, 27 Tai-Ping Road, Beijing, 100850, China; Tel.: 86-10-6816-9363; Fax: 86-10-6821-1656, E-mail: keliangliu55@126.com.

S.J.: Key Laboratory of Medical Molecular Virology of Ministries of Education and Health, Shanghai Medical College and Institute of Medical Microbiology, Fudan University, Shanghai 200032, China; Tel.: 86-21-54237673; Fax: 86-21-54237465; E-mail: shibojiang@fudan.edu.cn.

Table S1. Biophysical properties of the designed peptides[a]

Compd	Helicity (%)	*Tm* (°C)	Sedimentation coefficient (s)	Observed molecular mass (kDa)	Calculated molecular mass (kDa)
N36M	62	57	1.61	13.4	4.2[b]
N36MEK1	62	61	1.29	10.5	4.2[b]
N36MEK2	67	67	1.54	13.4	4.2[b]
$(N36M)_3$	82	>90	1.39	12.5	12.4
$(N36MEK1)_3$	89	>90	1.28	10.6	12.7
$(N36MEK2)_3$	100	>90	1.29	12.7	12.6
N36	27	undetectable			

[a] CD spectra of each designed peptide were monitored in PBS, pH 7.4. The final concentration of the peptide was 10 µM. Sedimentation equilibrium studies were performed at a concentration of 120 µM in PBS (pH 7.4) and a rotor speed of 60,000 rpm at 20 ºC. [b] The theoretical molecular mass for N-peptides in monomeric conformation.

Table S2. Designed peptides to assess the specificity of acyl transfer reaction

Compound	Sequence[a]
N36M(SBn)EK2	SEIV**KK** INNI<u>**E**</u>RA IEAQQKL LQLTVWG IKQLQAR IL
N36M(SBn)EK2a	SEIV**RK** INNI<u>**E**</u>RA IEAQQKL LQLTVWG IKQLQAR IL
N36M(SBn)EK2b	SEIV**KR** INNI<u>**E**</u>RA IEAQQKL LQLTVWG IKQLQAR IL
N36M(SBn)EK1	SEIV**KK** INNI<u>**E**</u>RA IEAQQHL LQLTVWG IKQLQAR IL
N36M(SBn)EK1a	SEIV**RK** INNI<u>**E**</u>RA IEAQQHL LQLTVWG IKQLQAR IL
N36M(SBn)EK1b	SEIV**KR** INNI<u>**E**</u>RA IEAQQHL LQLTVWG IKQLQAR IL

[a] Glu with side chain thioester is underlined

Table S3. Activity of N-trimers against T20-sensitive and T20-resistant strains[a]

NL4-3 mutant	T20 (EC_{50}, nM)	$(N36M)_3$ (EC_{50}, nM)	$(N36MEK1)_3$ (EC_{50}, nM)	$(N36MEK2)_3$ (EC_{50}, nM)
D36G	45.9 ± 7.4	20.2 ± 2.7	106 ± 16	19.4 ± 1.9
(36G)V38A	620 ± 103 (13)	14.5 ± 1.5 (0.7)	126 ± 31 (1.1)	21.7 ± 2.3 (1.1)
(36G)V38E/N42S	>2,000 (>43)	6.0 ± 3.7 (0.3)	22.2 ± 2.8 (0.2)	4.90 ± 2.9 (0.2)
(36G)V38A/N42T	539 ± 33 (12)	10.2 ± 1.3 (0.5)	71.0 ± 6.1 (0.7)	8.10 ± 1.2 (0.4)

[a] Compounds were tested in triplicate, and the data are presented as the mean ± standard deviation. The values in parentheses indicate relative changes (n-fold) in the EC_{50} compared with the EC_{50} in the presence of the D36G substitution. HIV-$1_{NL4-3(D36G)}$ is a T20-sensitive strain, and the others are T20-resistant strains.

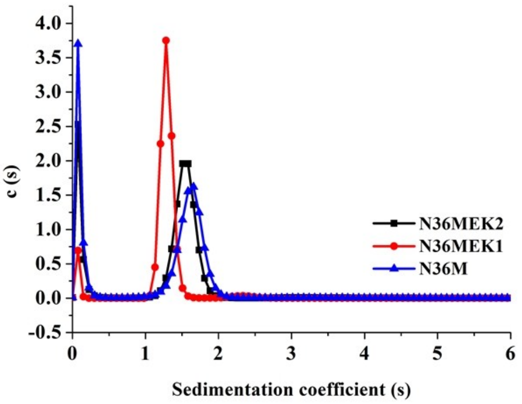

Figure S1. Sedimentation velocity analysis of N36MEK2, N36MEK1 and N36M

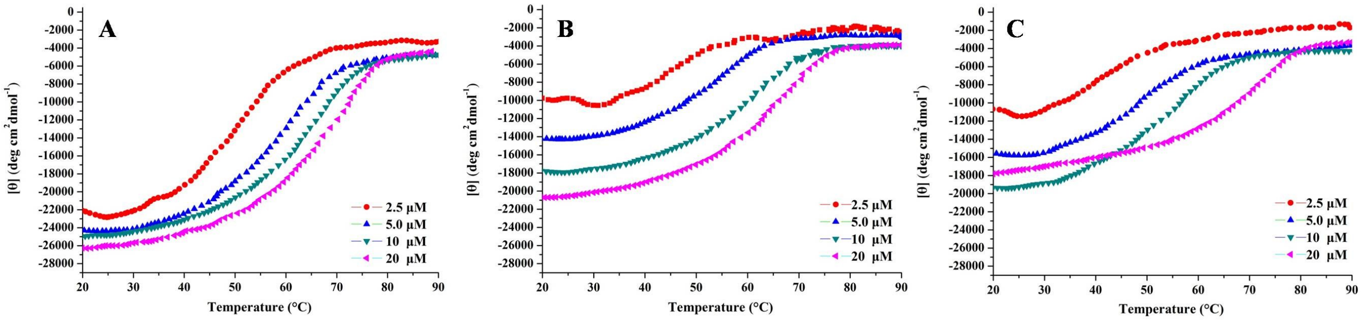

Figure S2. The thermostability of (**A**) N36MEK2, (**B**) N36MEK1 and (**C**) N36M at different concentrations in PBS were determined using CD spectroscopy.

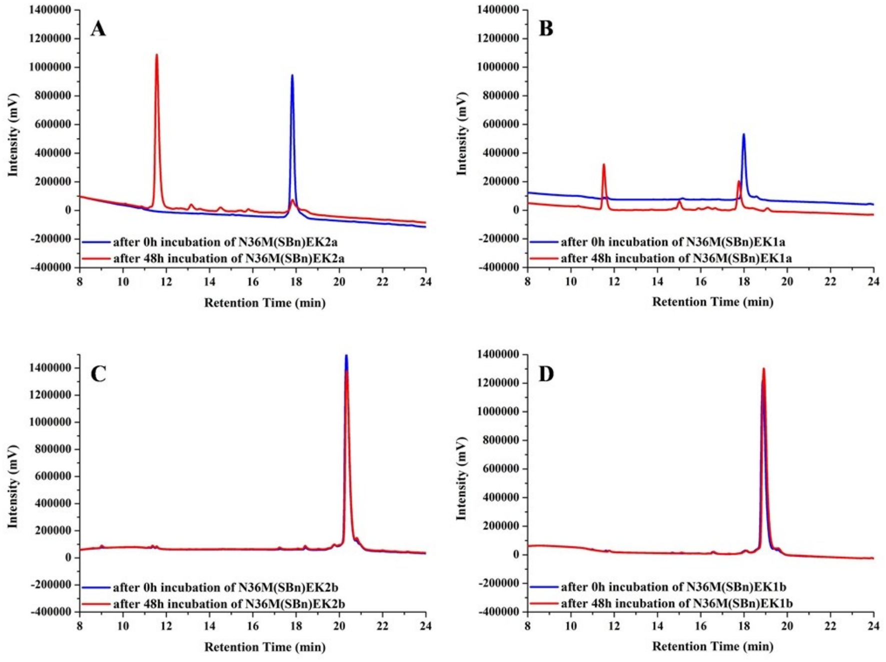

Figure S3. (**A-B**) RP-HPLC traces for the acyl transfer reaction of N36M(SBn)EK2a and N36M(SBn)EK1a at t=0 and 48 h. RP-HPLC traces for Lys-Glu ligation of (**C**) N36M(SBn)EK2b and (**D**) N36M(SBn)EK1b at t=0 and 48 h.

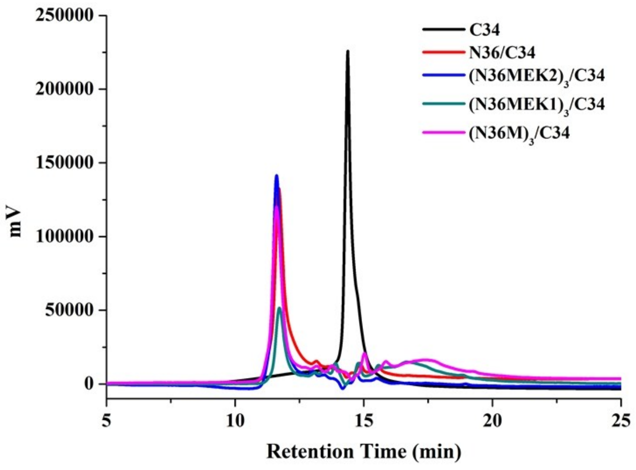

Figure S4. Determination of specific interactions between the N-peptides and C34 using SE-HPLC analysis.

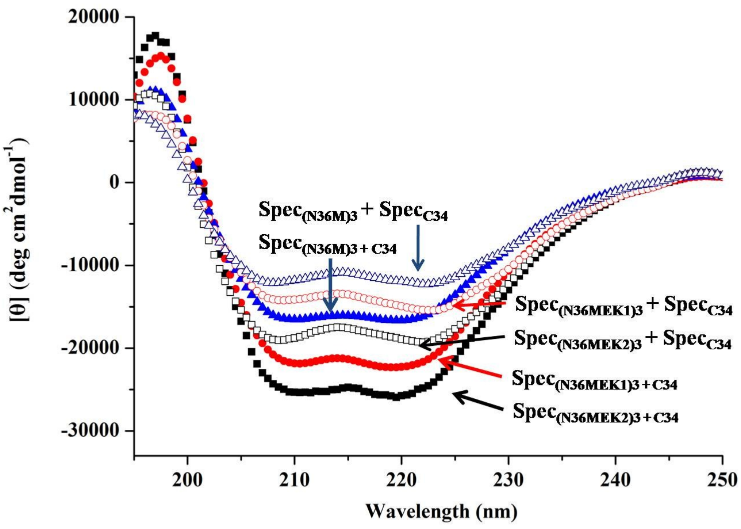

Figure S5. C34 and covalently stabilized N-trimers show interaction in solution. CD spectrum of peptide mixtures ($Spec_{N+C}$, solid symbols) and the sum of the spectra of the related isolated peptides ($Spec_N$ + $Spec_C$, open symbols) are shown for comparison. The covalently stabilized N-trimers-C34 interaction induces more α-helix structure than the sum of the single peptides. Final concentration of each peptide in PBS is 10 μM.

SI Methods

Peptide synthesis. Peptides were synthesized using a Liberty^{TM} automated microwave peptide synthesizer (CEM Co., Matthews, NC) with a standard solid-phase N-(9-fluorenyl)methoxycarbonyl (Fmoc) chemistry protocol. All protected amino acids used were purchased from GL Biochem Ltd. (Shanghai, China). Rink Amide resin (0.38–0.45 mmol/g, Nankai Hecheng S&T Co. Ltd., Tianjin, China) was used. Coupling of the amino acids was achieved using O-benzotriazol-1-yl-N,N,N',N'-tetramethyl-uronium hexafluorophosphate (HBTU, GL Biochem, Shanghai, China) and diisopropylethylamine (DIEA, Acrose) as an activator and an active base, respectively, in N,N-dimethylformamide (DMF) solution. The Fmoc protection group was removed using 20% piperidine/DMF. Between every coupling or Fmoc removal, the resin was washed five times with DMF and three times with dichloromethane (DCM). The carboxyl termini were amidated upon cleavage from the resin, and the amino termini were capped with acetic acid anhydride. For peptides possessing a side chain thioester, Fmoc-L-glutamic acid O-allyl ester [Fmoc-Glu(OAll)-OH] was used at the thioester-modified site. After all amino acids had coupled on the resin in the peptide synthesizer, the O-allyl group was removed manually by 1 eq tetrakis(triphenylphosphine)palladium with 10 eq 5,5-dimethyl-1,3-cyclohexanedione as scavenger in DCM/THF(1:1) solution. Then the resin was washed five times with 0.5% DIEA in DMF and five times with 1 M sodium diethyldithiocarbamate in DMF. 4 eq 1-Ethyl-3-(3-dimethyllaminopropyl)carbodiimide hydrochloride (EDC) and 4 eq benzyl mercaptan were added to the resin for thioester formation. The peptides were cleaved from the Rink Amide resin and deprotected with Reagent K, which contained 82.5% trifluoroacetic acid (TFA), 5% thioanisole, 5% m-cresol, 5% water, and 2.5% ethanedithiol. The crude peptide products were precipitated with cold diethyl ether, lyophilized, and purified by preparative reverse-phase high-performance liquid chromatography (HPLC) using a Waters preparative HPLC system (PrepLC 4000): gradient elution of 30–50% solvent B in solvent A (0.1% TFA in H_2O, solvent A; 0.1% TFA in 70% CH_3CN/H_2O, solvent B) over 60 min at 16 mL/min on a Waters X-bridge C8, 10 µm, 19.5 mm × 250 mm column. Analytical RP-HPLC was performed on a RP-C8 column (Zorbax Eclipse XDB-C8, 5 µm, 4.6 mm × 150 mm) with gradient elution of 5–100% solvent B in solvent A over 25 min at a flow rate of 1 mL/min. Compounds were detected by UV absorption at 210 nm with a Shimadzu SPD-10A detector. All peptides were purified to >95% purity. The molecular weight of the peptides was confirmed by matrix-assisted laser desorption/ionization-time of flight mass spectrometry (MALDI-TOF-MS; Autoflex III, Bruker Daltonics).

Isopeptide bond formation (interchain acyl transfer reaction). In brief, the purified thioester-peptide precursor (1.0 equiv., 0.5 µmol) was dissolved in 1 mL of $PBS/H_2O/CH_3CN$ (3:5:2 v/v) and stirred at room temperature for 24 h. The reaction was monitored by analytical RP-HPLC and confirmed by MALDI-TOF-MS.

Cell-cell fusion assay. Cell-cell fusion assays were performed as described previously.[1] HL2/3 cells, which stably express HIV Gag, Env, Tat, Rev, and Nef proteins, as well as TZM-bl cells, which stably express large amounts of CD4 and CCR5, were obtained from the NIH AIDS Reference and Reagent Program (contributed by Drs. Barbara Felber and George Pavlakis and Drs. John C. Kappes and Xiaoyun Wu, respectively). TZM-bl cells (2.5×10^4/well) and HL2/3 cells (7.5×10^4/well) were coincubated in 96-well plates (Corning Costar) at 37°C in 5% CO_2 in the presence of different concentrations of inhibitors. After incubation for 6–8 h, the medium was aspirated, the cells were washed and lysed, and luciferase activity was measured using the Luciferase Assay System (Promega, Madison, WI) on a SpectraMax M5 plate reader (Molecular Devices, Sunnyvale, CA).

HIV-1 infection assay. To measure the inhibitory activity of the peptides on infection of R5 primary HIV-1 isolates, US4/GS 007 (subtype B), 93IN101 (subtype C), and 92TH009 (subtype A/E), 50 µl of an inhibitor at graded concentration in triplicate was incubated with the same volume of a primary HIV-1 isolate at 100 $TCIID_{50}$ at 37 °C for 30 min, followed by addition of 100 µl of CEMx174 5.25M7 cells (5×10^5/ml). After incubation at 37 °C overnight, the culture medium was replaced with fresh medium without inhibitor. On day 7 postinfection, the culture supernatants were collected for measuring p24 by ELISA, and the percent inhibition and EC_{50} values were calculated using Calcusyn software as described above.[2]

Cytotoxicity assay. The *in vitro* cytotoxicity of the peptides towards the virus target cells (MT-2) was measured by the XTT assay. Briefly, 100 µL of each peptide at graded concentrations was added to an equal volume of cells (5×10^5/mL) in wells of a 96-well plate. After incubation at 37°C for 4 days, 50 µL of XTT solution (1 mg/mL) containing 0.02 µM phenazinemethosulfate were added. After 4 h, the absorbance at 450 nm (A_{450}) was measured with an ELISA reader. The CC_{50} values were calculated using CalcuSyn software.

Circular dichroism (CD) spectroscopy. Lyophilized N-peptides were resuspended in ddH_2O (pH 7.0) at a concentration of approximately 1 mg/mL. All the N-peptides, C-peptides, and the mixture of the N- and C-peptides were diluted in PBS (pH 7.4) to a final concentration of 10 µM, respectively. The individual peptides and their mixtures were incubated in a water bath set at 37 °C for 0.5 h before testing. CD spectra were acquired on an MOS-450 system (BioLogic, Claix, France) using the following parameters: band width, 4.0nm; resolution, 0.1 nm; path length, 0.1 cm; response time, 4.0 s; and scanning speed, 50 nm/min. For CD thermal denaturation analysis, the temperature was controlled by a BioLogic TCU250 system, and CD spectra were monitored at 222 nm from 15 ºC to 90 ºC at a scan speed of 2 ºC/min.

Sedimentation velocity analysis (SVA). All measurements were performed on a ProteomelabTMXL-A/XL-I analytical ultracentrifuge (Beckman Coulter, Fullerton, CA, USA) at 20 ºC, as described previously. In brief, three-channel cells were used with an An-60 Tirotor. Lyophilized N-peptides were resuspended in ddH_2O (pH 7.0) at ~1 mg/mL. The individual proteins and their mixtures with an equimolar amount of C34 peptide were prepared in PBS and incubated at 37 ºC for 30 min. All samples were prepared at a final concentration of 120 µM and were initially scanned at 3000 rpm for 10 min to identify the appropriate wavelength for data collection. Data were collected at 60,000 rpm at a wavelength of 280 nm. Sedimentation coefficient distribution, c (s), and molecular mass distribution, c (M), were calculated using the SEDFIT program.

Native polyacrylamide gel electrophoresis (N-PAGE). Tris-glycine gels (12%) and a BayGene Mini Cell were used for N-PAGE analysis. N-peptide in water was mixed with C-peptide in PBS and incubated at 37 ºC for 30 min (final concentration of N- and C-peptide, 120 µM). The samples were mixed 1:1 with 2 × Tris-glycine native sample buffer (BioRad, Hercules, CA, USA) and then loaded onto the gels. Gel electrophoresis was carried out at a constant voltage of 120 V at room temperature for 2.5 h, and the gel was subsequently stained with Coomassie blueR250.

Tricine-sodium dodecyl sulfate-polyacrylamide gel electrophoresis (Tricine-SDS-PAGE). Polyacrylamide gels (12%) and a BayGene Mini Cell were used for Tricine-SDS-PAGE. The cathode buffer consisted of 0.1 M Tricine, 0.1 M Tris, and 1% SDS, and the anode buffer was 0.2 M Tris. After incubation at 37 °C for 0–24 h, the samples were mixed with Tris-SDS-glycine sample buffer (Invitrogen, Carlsbad, CA) at a ratio of 1:1 and then loaded onto the gels (20 μL/well). Gel electrophoresis was first carried out at a constant voltage of 60 V at room temperature for 1 h and then carried out at a constant voltage of 120 V at room temperature for 2 h. The gel was then stained with Bio-Rad Bio-Safe Coomassie Stain.

Binding assays by size-exclusion chromatography. C34 was mixed with N-peptides (final concentration = 0.20 mM) at a molar ratio of 1:1 in PBS (pH 7.4) and incubated at 37 °C for 30 min. The peptide or peptide mixture (20 μL) was applied to a Phenomenex BioSep-SEC-s2000, 300 mm × 7.80 mm HPLC column equilibrated with PBS (pH 7.4) and eluted at 0.6 mL/min, and the fractions were monitored at 210 nm. Column pressure was 3.5 Mpa.

Metabolic stability

Qualification assay. Chromatographic analyses were performed using an ODS-C8 column (5 μm, 100 mm × 2.0mm ID) kept at ambient temperature. The mobile phase was composed of acetonitrile-water-formic acid in the ratio of 60:40:0.1 (v/v/v) at a flow rate of 0.3 mL/min. The sample injection volume was 10 μL. Acetonitrile was HPLC grade, and other chemical reagents and solvents were analytical grade. A Thermo TSQ Quantum Discovery MAX triple–quadruple tandem mass spectrometer equipped with ESI source (San Jose, CA) and Surveyor LC pump were used for LC-MS/MS analysis. Data acquisition and data processing were performed by using Xcalibur software and LCQuan 2.0 data analysis program (Thermo Finnigan), respectively. Optimized MS parameters were as below: 4800 V spray voltage, 10.0 psi sheath gas pressure, 1.0 psi auxiliary valve flow, and 300 °C of capillary temperature. When running collision-induced dissociation (CID), the pressure was set to 1.5 mTorr. The selected reaction monitoring (SRM) mode was used for $(N36M)_3$ while the selected ion monitoring (SIM) mode was preformed for T20. The following transitions were recorded: *m*/*z* 1555.6→1590.1 for $(N36M)_3$, *m*/*z* 1123.7 for T20.

Preparation of Samples. By use of a simple protein precipitation method, a test compound was extracted from rat plasma. Plasma samples for standard curves were prepared by spiking 100 μL of rat plasma with 100 μL of various concentrations of each test compound ranging from 0.1 to 50 μg/mL in H_2O. To each tested plasma sample (100 μL), 100 μL ethanol was added. After the mixture was vortexed and centrifuged at 18000*g* for 10 min, the supernatant was transferred to auto-sampler vials, and 10 μL of the supernatant was injected into the LC-MS/MS system for analysis.

Assessment of in Vitro degradation. Three male Sprague-Dawley rats (250 ± 20 g) were anesthesia, sacrificed by collecting blood via abdominal aorta. The liver and kidneys were quickly removed from each rat, washed in ice-cold 0.9% sodium chloride solution, weighed and cut into small pieces, diluted with 4 mL of ice-cold Tris–HCl buffer (50 mM, pH 7.4) per 1 g of tissue. For each tissue, the samples from the three rats were pooled and homogenized using a Teflon digital homogenizer. The homogenates were centrifuged (9,000 *g*) for 20 min at 4°C. Test compounds were added to the homogenates at a final concentration of 10 μg/mL, and then the reaction was initiated by heating at 37°C in a water bath. The samples (100 μL) were withdrawn at different time intervals (0, 30, 60 and 120min) and tested as described above.

MALDI-TOF-MS of designed N-peptides

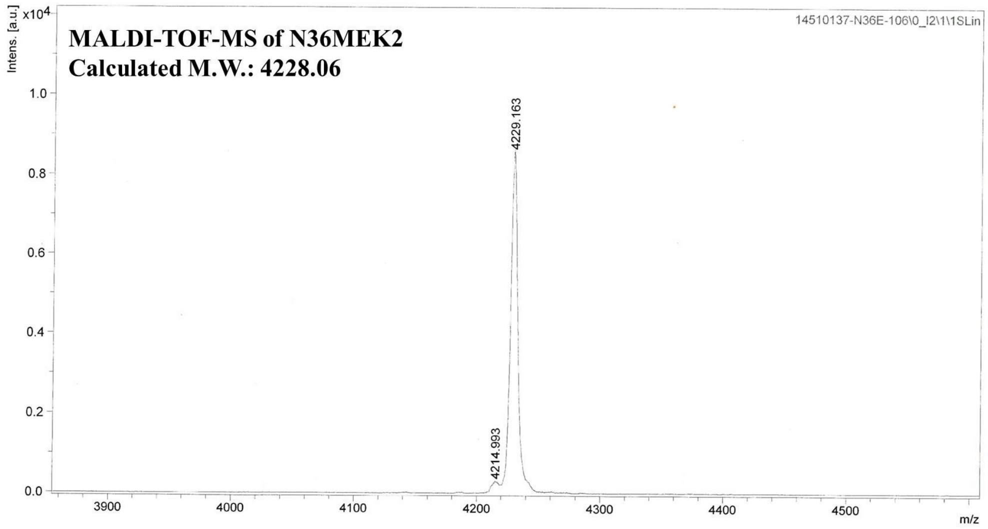

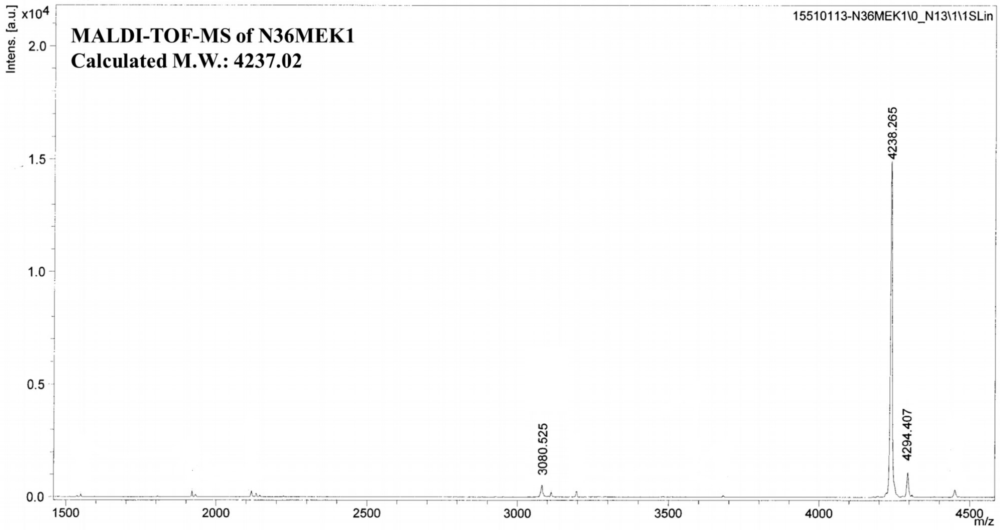

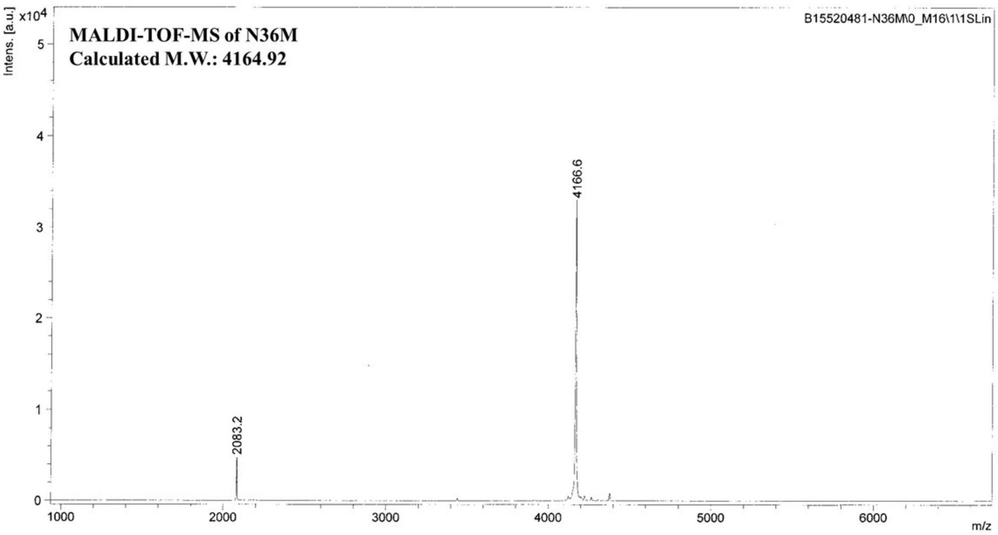

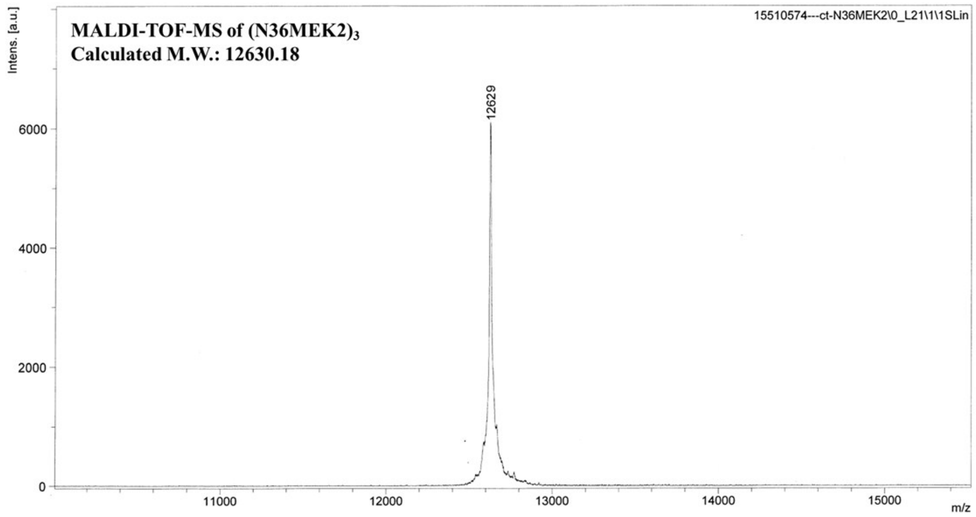
MALDI-TOF-MS of (N36MEK2)$_3$
Calculated M.W.: 12630.18
15510574---ct-N36MEK2\0_L21\1\1SLin
Intens. [a.u.]
12629
6000
4000
2000
0
11000
12000
13000
14000
15000
m/z

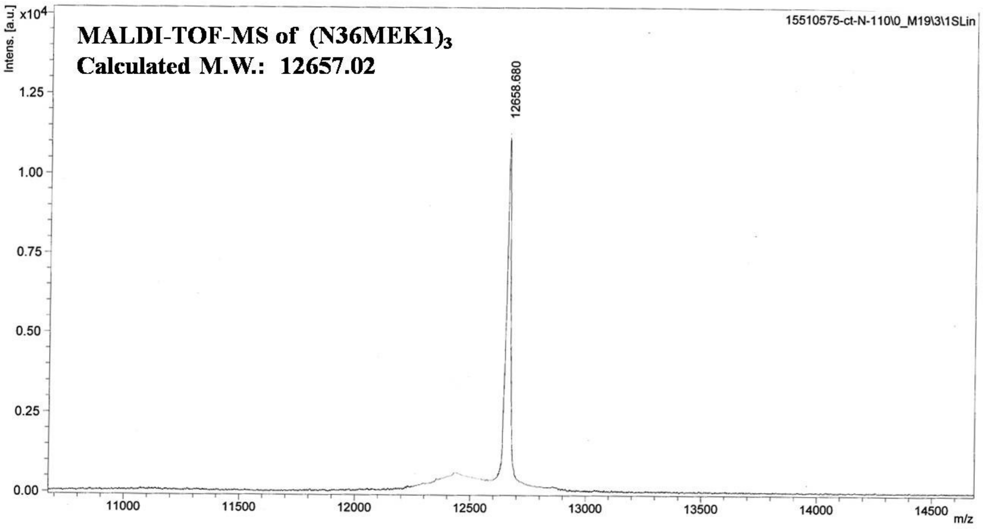
MALDI-TOF-MS of (N36MEK1)$_3$
Calculated M.W.: 12657.02
15510575-ct-N-110\0_M19\3\1SLin
Intens. [a.u.]
x10^4
12658.680
1.25
1.00
0.75
0.50
0.25
0.00
11000
11500
12000
12500
13000
13500
14000
14500
m/z

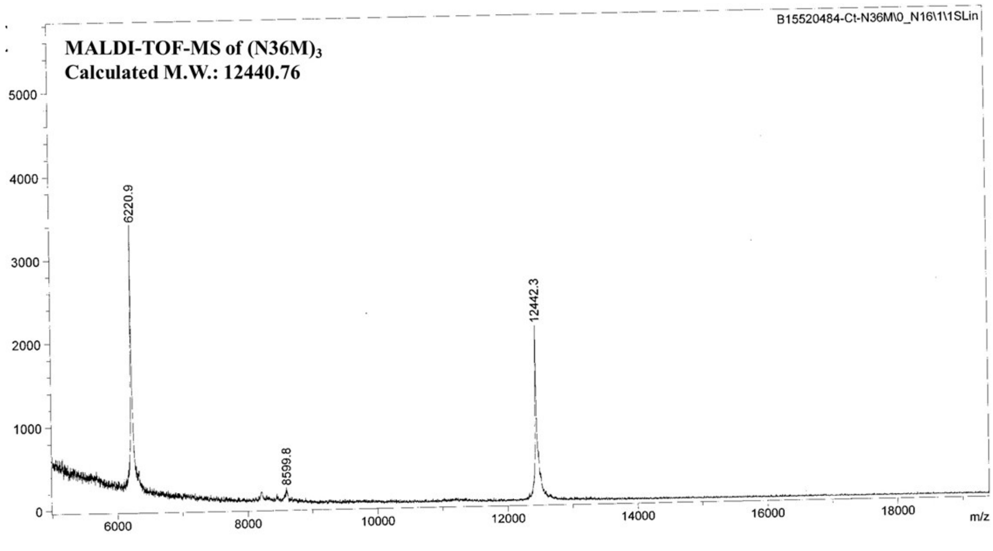
MALDI-TOF-MS of (N36M)$_3$
Calculated M.W.: 12440.76
B15520484-Ct-N36M\0_N16\1\1SLin
6220.9
8599.8
12442.3
5000
4000
3000
2000
1000
0
6000
8000
10000
12000
14000
16000
18000
m/z

References

(1) Wang, C.; Shi, W. G.; Cai, L. F.; Lu, L.; Wang, Q.; Zhang, T. H.; Li, J. L.; Zhang, Z. Q.; Wang, K.; Xu, L.; Jiang, X. F.; Jiang, S. B.; Liu, K. L. *J Med Chem* **2013**, *56*, 2527.

(2) Wang, C.; Shi, W. G.; Cai, L. F.; Lu, L.; Yu, F.; Wang, Q.; Jiang, X. F.; Xu, X. Y.; Wang, K.; Xu, L.; Jiang, S. B.; Liu, K. L. *J Antimicrob Chemoth* **2014**, *69*, 1537.
